# Screening for Dyslexia Using Eye Tracking during Reading

**DOI:** 10.1371/journal.pone.0165508

**Published:** 2016-12-09

**Authors:** Mattias Nilsson Benfatto, Gustaf Öqvist Seimyr, Jan Ygge, Tony Pansell, Agneta Rydberg, Christer Jacobson

**Affiliations:** 1 Department of Clinical Neuroscience, Division of Ophthalmology and Vision, Marianne Bernadotte Centre, Karolinska Institutet, Stockholm, Sweden; 2 Department of pedagogy, Linnaeus University, Växjö, Sweden; University of Muenster, GERMANY

## Abstract

Dyslexia is a neurodevelopmental reading disability estimated to affect 5–10% of the population. While there is yet no full understanding of the cause of dyslexia, or agreement on its precise definition, it is certain that many individuals suffer persistent problems in learning to read for no apparent reason. Although it is generally agreed that early intervention is the best form of support for children with dyslexia, there is still a lack of efficient and objective means to help identify those at risk during the early years of school. Here we show that it is possible to identify 9–10 year old individuals at risk of persistent reading difficulties by using eye tracking during reading to probe the processes that underlie reading ability. In contrast to current screening methods, which rely on oral or written tests, eye tracking does not depend on the subject to produce some overt verbal response and thus provides a natural means to objectively assess the reading process as it unfolds in real-time. Our study is based on a sample of 97 high-risk subjects with early identified word decoding difficulties and a control group of 88 low-risk subjects. These subjects were selected from a larger population of 2165 school children attending second grade. Using predictive modeling and statistical resampling techniques, we develop classification models from eye tracking records less than one minute in duration and show that the models are able to differentiate high-risk subjects from low-risk subjects with high accuracy. Although dyslexia is fundamentally a language-based learning disability, our results suggest that eye movements in reading can be highly predictive of individual reading ability and that eye tracking can be an efficient means to identify children at risk of long-term reading difficulties.

## Introduction

Dyslexia is a neurodevelopmental reading disability that adversely affects the speed and accuracy of word recognition, and as a consequence, impedes reading fluency and text comprehension. It is commonly estimated to affect between 5 and 10 percent of the population. Such estimates, however, depend on the definition and criteria used for diagnosis. Since reading ability is a skill that falls along a continuum, dyslexia is best considered a difficulty along this continuum with no clear-cut or absolute limit. Thus, it is not possible to specify exactly how common dyslexia is, other than in relation to an approximate limit of what can be considered normal reading ability. This relative uncertainty, however, does not disprove the reality of dyslexia; there is good evidence for its neurological basis [1–3]. Rather, it reflects the fact that dyslexia occurs in varying degrees of severity, and that, ultimately, a subjective cutoff must be set on a continuous variable in order to diagnose the disability [4–7].

Although the causes of dyslexia are still not fully understood, and definitions and terminology vary, it is generally agreed that children who fail to acquire reading skill at a normal rate need careful monitoring and support during the early years of school. Early identification and professional support is the most effective form of intervention for children with pronounced reading difficulties, and it is hazardous to wait until children are formally diagnosed with dyslexia before assisting their needs [7–9]. In Sweden, where this study was conducted, the average age at which dyslexia is diagnosed is 13 years [10]. By that age, it is not only very difficult to catch up to grade level in reading, the problems then usually interfere with overall school performance and cause psychological and emotional distress, manifested by low self-esteem, lack of motivation and depression [11–13].

Fast, systematic and automated screening methods based on objective measurements of reading may help identify individuals at risk of dyslexia during the early school years. Current methods, however, are limited in that they only measure individual cognitive skills that natural reading depends upon, but say little about their interplay and function in actual reading. Invariably, these tests require the subject to produce some explicit response, typically under time pressure, such as marking the word boundaries in sequences of words without interword spaces, matching target words to corresponding pictures, or reading aloud pronounceable nonsense words of increasing difficulty. The outcome measure–the proportion of correct responses–gives an estimate of performance on a particular task related to reading, but does not reflect the actual process of reading as it naturally occurs.

To overcome this limitation, we investigate the use of eye tracking during reading as a means for identifying children at risk of dyslexia and long-term reading difficulties. By tracking eye movements during reading, we are able to follow the reading process as it occurs in real-time and obtain objective measurements of this process as a whole. The data being sampled provide a next to continuous record of reading that reflects both the speed and accuracy of the processes involved [14–18]. Importantly, this mode of measurement requires no overt response extraneous to the reading process itself and thus makes it possible to assess reading performance without placing additional task demands on the subject. As such, this approach differs in important ways from the screening methods currently in use. Tests that involve performing a task by hand, for example, require subjects to engage motor skills beyond those involved in natural reading, which in turn may influence individual performance and confound results. On the other hand, tests based on pronouncing words out loud require manual assessments that are sensitive to subjective judgements and interrater variability, which easily introduce inconsistencies in the results.

Although it has long been known that the eye movements of dyslexic readers are different from those of typical readers, previous research has focused almost exclusively on identifying group-level differences [19–24]. Here we show, using machine learning and predictive modeling, that it is possible to move from group-level descriptions to individual-level predictions with high sensitivity and specificity, which is a first step towards making eye tracking a viable screening method. Using statistical cross-validation techniques on a sample of 97 high-risk and 88 low-risk control subjects, we achieve a classification accuracy of 96% with balanced levels of sensitivity and specificity. We also compare the relative importance of different eye movement features and identify some critical features that differentiate high-risk and low-risk subjects. Overall, our findings suggest that eye tracking combined with machine learning can be used to develop fast, objective and accurate screening models useful for identifying school children at risk of dyslexia.

## Materials and Methods

### Participants

The experiments we report are based on eye tracking data from 185 subjects participating in the Kronoberg reading development project, a longitudinal research project on reading development and reading disability in Swedish school children running between 1989 and 2010 [25,26]. From an original cohort of 2165 individuals attending second grade (age 8–9), 103 subjects who had failed to develop word reading skills at a normal rate were first identified in 1989. This group of high-risk (HR) subjects consisted of 82 male subjects (7.7% of all males) and 21 female subjects (1% of all females). The inclusion criteria required that subjects (1) had Swedish as first language; (2) performed in the lower 5^th^ percentile of the full cohort on two standardized tests of word decoding; and, (3) experienced persistent problems in learning to read according to an independent assessment completed by the classroom teacher. Individuals with intellectual disability, at the time known as mental retardation, were excluded in the selection.

The selection of HR subjects did not involve any discrepancy-based definition of dyslexia. That is, inclusion did not require an apparent discrepancy between reading level and general cognitive ability or intelligence quotient (IQ). This is consistent with the predominant view of dyslexia today since such a requirement has been discredited by empirical evidence suggesting that dyslexia occurs across the range of intellectual abilities and represents the low end of a normal distribution of word reading ability [5,27,28].

A control group of low-risk (LR) subjects with average or above average word reading skills were pairwise matched to the HR subjects on sex, first language, school class, and non-verbal ability (Raven’s progressive matrices). Given the matching criteria, 90 pairwise matched controls could be identified, 70 male and 20 female subjects. Thus, 12 male subjects and 1 female subject in the HR group were not matched to any control subject.

The cognitive, educational and social development of LR and HR subjects was assessed at various intervals over the following 20 years, until 29 years of age. During this time, the negative long-term effects of the early manifested reading difficulties in the HR group have been extensively documented [25,26]. Follow-up studies show that word decoding and reading problems persisted for the large majority of subjects and significantly interfered with school performance, academic achievement and other domains of life (see S1 Text for a summary of follow-up studies). It is worth noting, however, that we do not know how many of the original HR subjects received an actual diagnosis of dyslexia later on. The main reason for this is that during the initial years of data collection the notion of dyslexia was still not well established in pedagogic practices in Sweden and very few individuals were diagnosed in general. As dyslexia diagnoses became more common over the years, most of the HR subjects in the study had already finished school which further reduced their likelihood of receiving a diagnosis.

While the subjects were attending 3^rd^ grade (age 9–10), eye movements were recorded as part of an ophthalmological examination that aimed to investigate whether there were any differences between the two groups in terms of basic visual and oculomotor functions [29,30]. While some minor differences were reported, it was concluded that these differences most likely reflected secondary effects of the cognitive difficulties that the HR subjects experienced with language processing, rather than inherent visual or oculomotor deficits. For our present experiments, we use eye movement recordings made while the subjects were reading a short natural passage of text adapted to their age. Recordings were available for 185 subjects, 97 HR subjects (76 males and 21 females) and 88 LR subjects (69 males and 19 females). Thus, from the original sample of 193 selected subjects, 6 HR subjects (6 males) and 2 LR subjects (1 male and 1 female) were not included in the experiments reported here.

The Kronoberg reading development project adhered to the principles of the Declaration of Helsinki. Written informed consent was obtained from the next of kin, caretakers, or guardians on behalf of the children enrolled in the study. At the time the project was initiated, there was no ethics committee to approve the study protocol, but such an approval was later obtained for the second part of the project in 2008 by the Regional Ethical Review Board at Linköping University (142–08).

### Apparatus and Stimuli

A goggle-based infrared corneal reflection system, Ober-2^TM^ (Formerly Permobil Meditech, Inc., Woburn, MA), was used to track eye position over time, sampling the horizontal and vertical position of both eyes at 100 Hz. Under well-controlled experimental conditions, the system afforded a spatial resolution of 5 minutes of arc along the horizontal axes, as per the manufacturer's specification. During recording, subjects were equipped with a pair of light-weight (80g), individually adjustable, head-mounted goggles in which four arrays of infrared transmitters and detectors were mounted, arranged in a square around each eye. A chin and forehead rest was deployed to minimize head movements and stabilize the viewing distance at 45 cm. Calibration was performed manually prior to each recording by setting the signal gain of each axis separately for each eye. Thus the gain for horizontal movements of the left eye was first set, then the gain for horizontal movements of the right eye and so on for vertical movements (Information on whether or not monocular occlusion was used during the calibrations is not available. We have reasons to assume it was but cannot confirm this.).

All subjects read one and the same text presented on a single page of white paper with high contrast. The text was distributed over 8 lines and consisted of 10 sentences with an average length of 4.6 words. By comparing the number of types to the number of tokens in the text we observed a type-token ratio ($TTR=\frac{n(type)}{n(token)}$) of 71.7%; a word variation index ($OVIX=\frac{\log\left(n(token)\right)}{\log\left(2-\frac{\log\left(n(type)\right)}{\log\left(n(token)\right)}\right)}$) of 46; and a word variation ratio ($OVR=\frac{\log\left(n(type)\right)}{\log\left(n(token)\right)}$) of 91.3%. The subjects were instructed to read the text silently and to answer three questions about its content afterwards. The questions mainly served to encourage the subjects to read for comprehension; the actual outcomes were not used in any step of our analysis.

### Eye Movement Analysis

**Fig 1:**
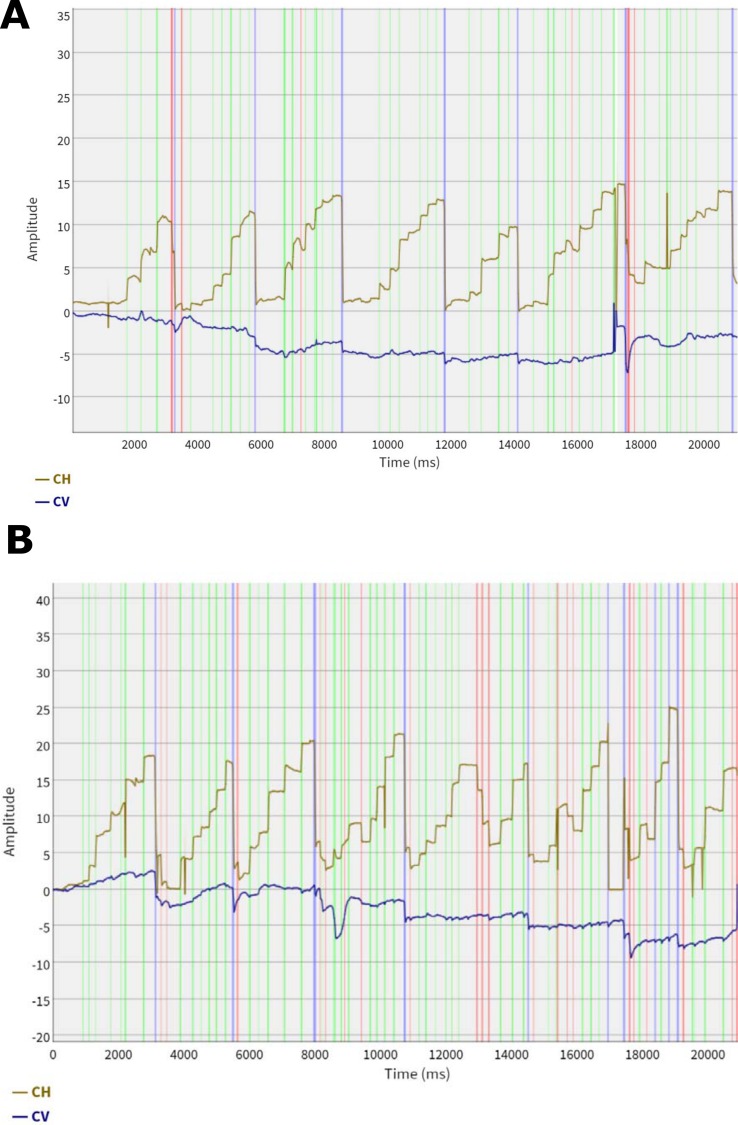
Example of eye movement analysis where the horizontal (CH) and vertical (CV) eye movement signal is plotted over time. Light green stripes represent saccades, light gray areas represent fixations. Light blue stripes represent sweeping movements (most commonly return sweeps) and red stripes represent transients. Plot **A** represents a subject from the HR group and plot **B** a subject from the LR group. The analysis was performed using a dynamic dispersion threshold algorithm based on the physiological properties of the foveal and parafoveal fields of vision. The algorithm analyzes the tracking signal sample by sample and switches between four mutually exclusive states: distortions, transients, fixations, and saccades. A distortion state is detected if the horizontal or vertical signal is missing for both eyes. A transient state is detected if the horizontal and vertical position is within a threshold distance of 0.5 degrees + signal noise (2.5 × RMS error of the last 25 samples) from the average of the samples in the current state. A fixation state is detected when the eyes have remained stable for at least 50 ms, and a saccade state when the eyes have moved beyond the threshold distance. Once a change of state is detected, the samples of the previous state are identified as a new event.

In order to identify fixation periods, saccadic movements and other types of events in the eye movement recordings, we first analyzed the raw recording signal of eye position over time using a dynamic dispersion threshold algorithm (Fig 1). On the basis of this analysis, we extracted eye movement features to use as input for training a classification model to discriminate between HR and LR subjects. Since we did not want to gear the feature extraction process towards specific assumptions regarding potential differences in eye movement behavior between the two groups, we strived to make a broad, systematic and unbiased selection of features that preserved as much as possible of the original eye movement signal. Hence, we defined a simple set of low-level features that ranged over both fixation and saccadic events. To avoid excessive data reduction, fixations and saccades were not further aggregated to composite word-based measures.

Saccades were divided into progressive (left-to-right) and regressive (right-to-left) movements and fixations were defined accordingly, depending on the direction of the preceding saccade. For each type of fixation and saccade, we defined parameters measuring (1) the duration of the event; (2) the distance spanning the event; (3) the average eye position during the event; (4) the standard deviation of the average position; (5) the maximum range between any two positions; and, (6) the accumulated distance over all subsequent positions. The parameters 2–6 were measured horizontally and vertically, and for both version and vergence, computed as the average position of the two eyes ((left + right)/2), and as the difference in the positions of the two eyes (left–right), respectively. Finally, the mean and standard deviation over each parameter distribution were computed, producing in total 168 features. The information contained in these features captures different quantitative properties of eye movements in reading, including their duration, amplitude, direction, stability and symmetry.

### Classification and Feature Selection

We trained maximum-margin classifiers using linear Support Vector Machines [31,32] (SVMs) with sequential minimal optimization [33,34]. This learning algorithm has previously been successfully applied to a wide range of classification problems, not least in bioinformatics where its use for drug discovery, biomarker identification, and development of new diagnostic tests has grown rapidly in recent years. All classifiers that we trained were evaluated with respect to their predictive performance. In other words, we assessed the classifiers’ ability to identify subjects, whose recordings were not used in fitting the model parameters to the data, as HR or LR subjects. All else equal, this provides an estimate of the extent to which the classifiers are able to predict HR/LR-status of future, previously unseen, test subjects on the basis of the subjects’ eye movements during reading. The predictive performance was assessed using a 10-fold stratified cross-validation procedure, repeated 100 times in order to stabilize the estimates across different random partitions of the dataset.

In order to remove irrelevant and redundant eye movement features, which may degrade the performance of the learning algorithm, we applied an automatic feature selection method known as recursive feature elimination (SVM-RFE) [35] during training. Removing features with little or no predictive information not only reduces the level of noise in the parametrized classifiers, but also facilitates a better understanding of which eye movement features aggregate to give the best predictive performance. Importantly, however, if the feature selection process is invoked on the whole dataset in one single step prior to training, it would result in a biased and overly optimistic estimate of the predictive performance [36,37]. To avoid such a situation, the feature selection algorithm was executed within each training fold, thus repeated in its entirety 10 × 100 times. To train classifiers using recursive feature elimination (SVM-RFE) we initiated the process with the full set of features, built the classifier on the training data and ranked the features by the square of the weight assigned by the SVM. The lowest-ranked feature was then removed and the training process repeated, eliminating one feature at a time until all features were exhausted and a complete ranking of the original feature set was obtained. In order to find the best classification model we then evaluated the classification accuracy of all possible classifiers as a function of the number of top-ranked features selected during training.

To demonstrate better than chance performance, all classifiers were tested against identical versions trained with randomly shuffled class labels, so called *Y*-randomization. To train these classifiers, we let half of the subjects in the HR and LR group in the current training fold swap places, that is, the class labels HR and LR were randomly permuted while the feature values for the same subjects remained unchanged. The classifier built on the randomized training data was then used to predict the non-randomized test set with the true labels preserved. Having purposely introduced noise in the training data which obscures the actual relationship between the target class and features, we expect the resulting classifiers to perform no better than chance on average.

**Fig 2:**
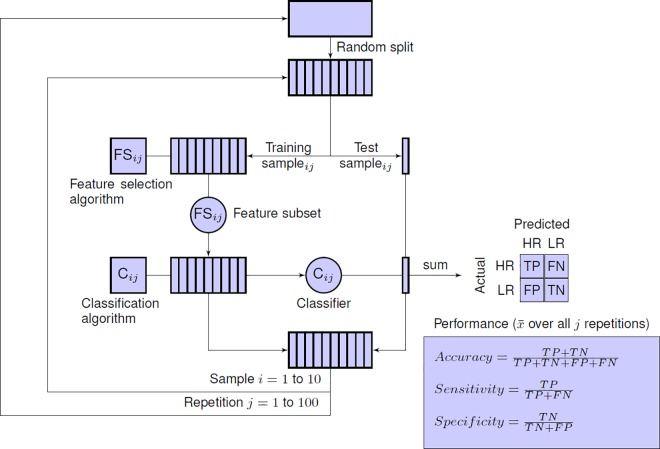
Experimental test protocol based on repeated cross-validation with internal feature selection. The entire dataset is randomly divided into 10 subsets, setting aside one subset (10% of all subjects) as a test sample and the remaining nine subsets (90% of all subjects) as a training sample. A feature selection algorithm is applied on the training sample to select a subset of *n* features. Using this feature subset, a classification algorithm is applied on the training sample, producing a parametrized classifier as output. This classifier is then used to classify the subjects in the test sample and the predicted results are compared to the actual identity (HR or LR) of the test subjects. This step is iterated 10 times, with a different training and test set for each iteration. After one completed run of 10-fold cross validation, each subject in the entire dataset has been tested exactly once, while we still have maintained a strict separation between training and test subjects. To reduce the variance of the cross-validated performance estimate, the whole process is repeated 100 times with different initial random splits of the original dataset. The final estimate of the expected predictive performance is calculated by averaging the cross-validation performance over all 100 repetitions. This estimate represents the expected prediction accuracy of the final model. The final model–the one we would deploy in practice–is the classifier we would build from the entire dataset using feature selection method *m* to select *n* features.

To assess the ability of the recursive feature elimination algorithm to select predictive features for classification, the performance of all classifiers was compared to that obtained by simply selecting features at random. Thus, to train classifiers with *n* randomly selected features, we sampled *n* features from the full feature set uniformly at random in each training fold. The classifier was then trained on this feature subset and subsequently applied to predict the examples in the test set. All experiments were implemented using the same training and test protocol (Fig 2).

## Results

### Classification Accuracy

**Fig 3:**
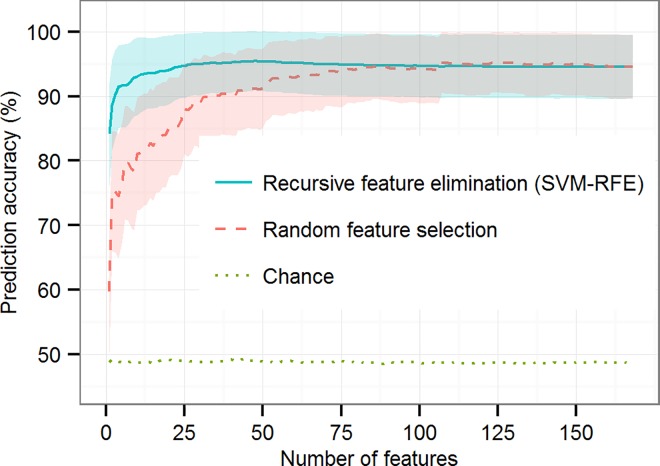
Prediction accuracy as a function of the numbers of features selected during training. Accuracy is shown for classifiers based on recursive feature elimination (solid blue line), random feature selection (dashed red line), and chance (dotted green line). Chance-level accuracy is based on Y-randomization of training data. Accuracy is the percentage of correctly identified HR and LR subjects averaged over 100 × 10-fold cross-validation. Maximum accuracy, 95.6%, (± 4.5%), is obtained using recursive feature elimination to select 48 features from the original feature set of 168 features. Shaded regions indicate mean ± 1 standard deviation over the 100 repetitions. Performance at chance level, averaged over the feature subset sizes, is 49.3%.

Among all classifiers, the best classification accuracy observed was 95.6% ± 4.5%, obtained by using SVM-RFE to select 48 features from the original feature space (Chance: 49.1% ± 13%, corrected resample t-test, *p* < 0.01; Random feature selection: 91.1% ± 6.1%, corrected resample t-test, *p* < 0.05) (Fig 3). This model also produced the smallest sample standard deviation and an optimal balance between sensitivity (95.5% ± 4.6%) and specificity (95.7% ± 4.5%), which means that the classifier performed as well in identifying HR subjects as it did in excluding LR subjects. By using recursive feature elimination we were thus able to decrease the overall complexity of the classifier, effectively reducing the original feature space by 71%. This shows that an automatic feature analysis can be applied to select a few highly informative eye movement features useful for prediction and discard other ones.

Using random feature selection, the best classification accuracy achieved was 95.3% ± 4.6% (Sensitivity 95.2% ± 4.7%; Specificity 95.5% ± 4.5%), obtained by selecting 126 features randomly in each training fold (chance: 48.6% ± 13.2, corrected resample t-test, *p* < 0.01). This means that the best classifier trained with randomly selected features performed on a par with the best classifier based on recursive feature selection, but only reduced the original feature space by 25%, thus yielding an accurate but unnecessarily complex model. The accuracy of the classifiers based on recursive feature elimination and those based on random feature selection converged for feature subsets about half the size of the original feature set. This indicates that the effectiveness of SVM-RFE, over random feature selection, ceases at about that point, that is, when 50% or more of the original feature set is used to train the classifiers.

In line with our expectations, the Y-randomized versions of the classifiers performed significantly worse than all other models regardless of the size of the feature subset, yielding accuracies in line with those expected on the basis of purely random prediction. Thus, we found no evidence of chance correlation in the data that would drive the competitive performance of the other classifiers.

How do these results compare to the level of accuracy we can expect from traditional screening tests based on oral or written tests? First, it is important to note that such a comparison is difficult for a number of reasons, perhaps foremost because few of the documented tests provide estimates of the accuracy of individually predicted outcomes. Hence the expected accuracy, or sensitivity and specificity, of most screening tests in current use are not well known. But even among studies that do report such estimates, comparisons are highly problematic because of differences in the age range considered, the definition of dyslexia adopted and the rigor of the validation methodology used.

The accuracy of screening instruments that are administered to children before they have received formal reading instruction in school, up until about 6 years of age, is typically reported to range between 70–80%. Notably, however, the level of sensitivity and specificity tends to be highly imbalanced for any given test, which severely limits the practical use of most tests. For example, in Pennington et al [38] we find that tests of phonological awareness (PA) and rapid automatized naming (RAN) individually produced a high specificity of 93.5%, but low sensitivity of 41.5% and 42.7% respectively. Thus, even though both PA and RAN did very well in excluding children that did not develop dyslexia, the majority of children that actually did also went by undetected by these tests.

The accuracy of screening tests increases as children begin school and receive formal reading instruction. In second and third grade, the accuracy typically ranges between 80–90%. However, it appears that in order to obtain balanced levels of sensitivity and specificity, multiple tests must be administered that collectively measure a combination of different cognitive skills related to reading. But if several different tests must be administered, and each test manually assessed, the possibility to implement an efficient screening process with large numbers of school children is seriously compromised. This is potentially one of the barriers that prevent many schools from implementing routine screening for dyslexia today.

### Feature Analysis

**Fig 4:**
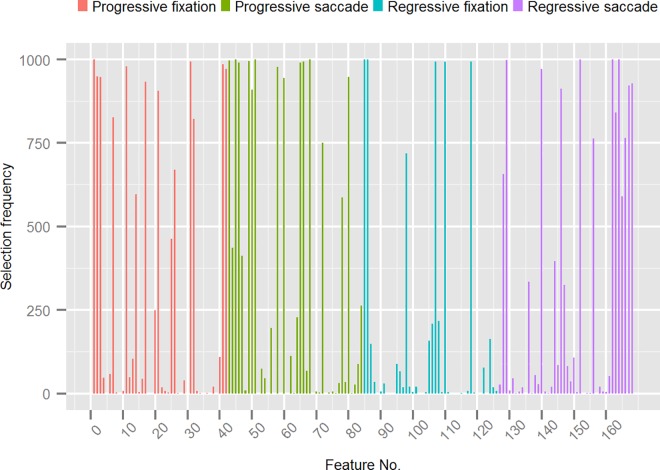
Frequency of features selected during training of the best performing classification model grouped by progressive/regressive fixation- and saccade features. The Y-axis shows the number of times a feature in the original feature set was selected by the recursive feature elimination algorithm (SVM-RFE) during the 10 x 100 cross-validation with internal feature selection. The X-axis shows the features (represented by their index in the dataset) grouped by progressive/regressive fixation- and saccade features.

Up to this point, we have only examined the output of the classification process, without specific consideration to the internal feature structure of the models that generated the output. In particular, we have yet no understanding of which eye movement features were more important than others for making accurate predictions. To better understand the relative importance of different features, we focused on the best performing classification model from the first experiment and analyzed the frequency with which different features were selected across the 1000 internal training folds (Fig 4). The more often a feature was selected, the more likely it contributes useful information that adds to the overall predictive accuracy of the classifier.

**Fig 5:**
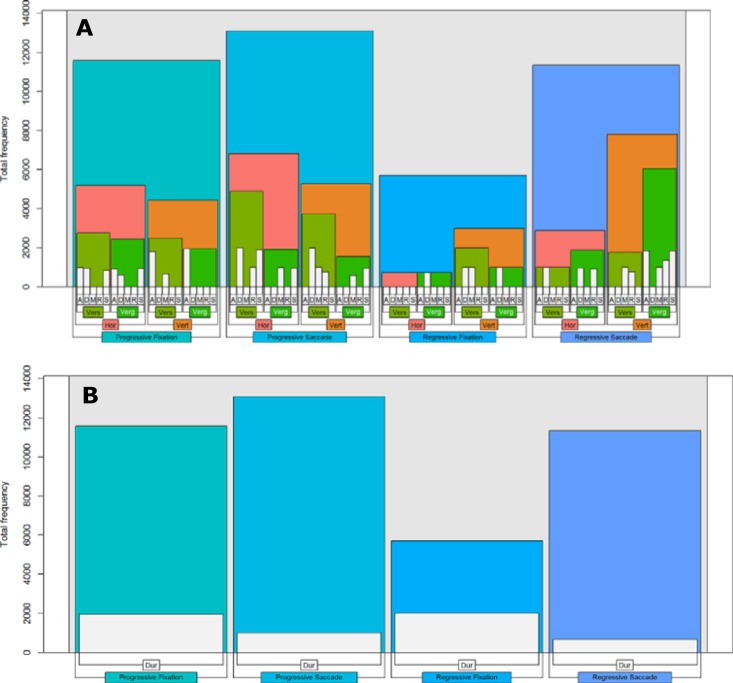
**Hierarchical breakdown of spatial (A) and temporal (B) features selected in more than 50% of training folds by the best performing classification model.** Forty-six features were selected in more than 500 (50%) training folds with a total accumulated selection frequency of 41721. Plot A and B show the breakdown of the features on this distribution. Spatial and temporal features are presented separately as they cannot be meaningfully nested within each other. In plot A, progressive/regressive fixation- and saccade features are broken down by horizontal (Hor) and vertical (Vert) axis, by version (Vers) and vergence (Verg) position, and by event parameter (A: accumulated distance, D: spanning distance, M: mean position, R: maximum range, and S: standard deviation of mean position). In plot B, progressive/regressive fixation- and saccade features are broken down by duration (Dur).

We found a large spread in the selection frequency of different features. Most of the original features were selected on few occasions and a few on most occasions, resulting in a large skew overall (min = 0, q_1_ = 2, median = 35, q_3_ = 660, max = 1000). Broken down by eye movement type, 24% of the selected features related to progressive fixations (min = 1, q_1_ = 8, median = 104, q_3_ = 907, max = 1000), 26% to progressive saccades (min = 1, q_1_ = 29, median, = 228, q_3_ = 963, max = 1000), 21% to regressive fixations (min = 1, q_1_ = 7, median = 34, q_3_ = 210, max = 1000), and 29% to regressive saccades (min = 1, q_1_ = 8, median = 56, q_3_ = 765, max = 1000). Forty-six features (27.4%) were selected in the majority of training folds, that is, in more than 500 training folds. Most of these features turned out to be rather stable across the folds (min = 588, q_1_ = 858, median = 971, q_3_ = 996, max = 1000), which indicates that they were robust to small random perturbations of the training data. For these most salient features, we would like to identify not only how they were distributed across progressive and regressive eye movements, but also across the horizontal and vertical axis, by version and vergence, and by individual fixation-saccade parameters. To achieve this, we constructed nested bar plots that show the hierarchic breakdown of the features into these variables (Fig 5).

Along the spatial dimension, we found that the majority of features relating to progressive fixations and saccades were calculated along the horizontal axis, whereas the majority of features relating to regressive fixations and saccades were calculated along the vertical axis. Thus it seems that for normal left-to-right reading, horizontally-based features tend to be more informative for the classifier than features along the vertical axis. When normal reading is interrupted, however, and the eyes regress to earlier parts of the text, features along the vertical axis appear to be more discriminative. Another difference observed concerns the distribution of features relating to progressive and regressive saccades. The majority of features relating to progressive saccades were calculated from the average of both eyes whereas the majority of features relating to regressive saccades were calculated from the difference between the left and right eye. Thus, version-based features appear to be more discriminative than vergence-based features for saccades that move the eyes forward, whereas the opposite is true for saccades that move the eyes back to previous parts of the text. We did not observe any particular differences in the distributional pattern of fixation and saccade parameters, which were roughly equally distributed among other variables. Along the temporal dimension we found that features relating to the duration of eye movements were more frequent for fixations than for saccades, which is expected given that the variability in fixation duration is known to be strongly associated with cognitive processing demands during reading.

**Fig 6:**
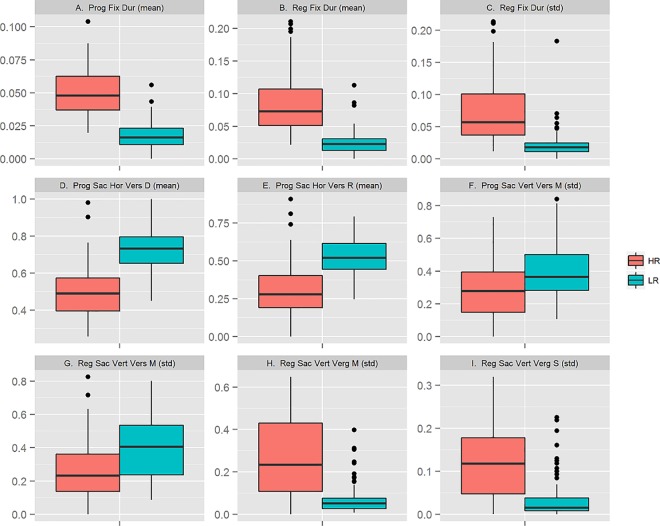
Box plots of features selected in 1000 (100%) training folds by the best performing classification model. The box plots show the distribution of values, normalized to range between 0 and 1, by feature and group HR (*n* = 97) and LR (*n* = 88).

Lastly, we examined the features with the highest frequency, that is, those that were constantly selected in all 1000 training folds. We found three features relating to the duration of fixations, three features relating to the distance, maximum range, and mean position of progressive saccades, respectively, and three features relating to the mean position and standard deviation of regressive saccades. We summarized each of these features by box plots, comparing the actual distribution of values between the HR and LR group (Fig 6).

We found that some of these features reflect previous experimental findings of differences in eye movements between dyslexic and non-dyslexic readers. For example, the mean duration of fixations, both progressive and regressive, was longer (higher median) in the HR group compared to the LR group (Plot A-B), and the distance spanning progressive saccades, as well as their maximum within-range, was shorter (lower median) in the HR group than in the LR group (Plot D-E). These differences likely reflect the underlying cognitive difficulties that the HR subjects experience in processing the words they read. In particular, the greater effort involved in decoding individual words results in longer fixation durations on average and an overall increase in fixation rate that decreases the length of saccades.

We also found features which reflected differences between the groups with respect to the spread around the mean vertical position of progressive and regressive saccades (Plot F-G), as well as in vertical vergence movements during regressive saccades (Plot H-I). These patterns are less intuitive to explain; understanding their significance for the predictive performance of the classifier requires further investigation. It may be noted, however, that a number of recent studies have reported differences between dyslexic and non-dyslexic readers in vergence and binocular coordination during saccades and fixations [24,39–41]. At present, though, the magnitude of these differences and their neurological basis are unclear.

## Discussion

Children with dyslexia often spend many years struggling in school before receiving appropriate professional support. Efficient screening methods that can be easily deployed in school settings are important instruments to counter this situation and facilitate earlier support to those at risk of long-term reading difficulties. It is unclear, however, whether the tests that have been developed to date serve the purpose they intend. The Swedish Council on Technology Assessment in Health Care (SBU) recently presented a systematic review of the scientific evidence for screening and diagnostic tests for children and adolescents with dyslexia [42]. According to this report, most of the tests in use today lack scientific support. A key concern, raised in the report, is that the existing tests are insufficiently evaluated with respect to their predictive validity, which makes it difficult to ascertain how useful the tests are in practice when applied to any given individual.

Here, we have investigated the use of eye tracking during reading as a screening method and demonstrated that it can produce individual-level predictions with high sensitivity and specificity in less than a minute of tracking time. In contrast to existing screening tests which rely on paper-and-pencil protocols, this method requires no written or verbal response and no manual assessment or grading in the traditional sense. The only response we measure is the eye movement signal and that itself is objective; it is neither right nor wrong according to some predefined criteria. Moreover, it seems probable that a screening test based on eye tracking may reduce the amount of stress that more traditional test methods impose, since subjects may be more likely to experience that they are engaged in a task by themselves rather than explicitly performing a task for someone else.

While we believe that our results show that eye tracking can be useful for screening of dyslexia, it is important to note that our approach is not driven by the assumption that dyslexia is caused by an intrinsic deficit in visual perception or oculomotor control. This is an important point because, historically, such deficits have been implicated as a cause of dyslexia [43–45]. Over the years, however, much research has made clear that dyslexia is a language-based disorder associated with a phonological deficit which compromises the ability to process written words and impedes reading comprehension [46,47]. In line with this view, the assumption we make is only that the ease or difficulty with which words are processed by the language system has an essentially immediate influence on eye movements during reading. A long line of research in cognitive psychology and psycholinguistics has shown this to be the case [48–50]. Thus, even though atypical eye movements in reading are only a secondary consequence of dyslexia, eye tracking may be an effective way to assess the processing demands that subjects experience during reading, and, by extension, a sound basis for developing predictive and automated models useful for screening. Similar approaches, based on tracking eye movements during free viewing of natural images and videos, have recently been developed and successfully applied to differentiate subjects with Parkinson’s disease, schizophrenia and autism spectrum disorders from control subjects [51–53].

Finally, it is important to stress that not all children who experience persistent difficulties in learning to read fit the same neuropsychological profile. It is well-established, for example, that there is considerable symptom overlap and a high rate of comorbidity between dyslexia, attention-deficit hyperactivity disorder (ADHD) and language impairment [54–55]. Moreover, it is also common to distinguish between different subtypes of dyslexia (e.g., surface *vs* phonological dyslexia). Therefore, diagnostic follow-up of a positive screening result is always necessary to gather a more comprehensive understanding of an individual’s cognitive profile, so that intervention strategies can be tuned to individual needs. Nevertheless, early identification of individuals in need of support is the first important step in this process. For this purpose, using eye tracking during reading may prove very useful.

## Supporting Information

S1 Text Summary of follow-up studies age 11–29. (DOCX)
