# Supplementary material for: Screening for Dyslexia Using Eye Tracking during Reading
Source: PLoS One. 2016 Dec 9;11(12):e0165508. doi: 10.1371/journal.pone.0165508 (PMC5147795; doi:10.1371/journal.pone.0165508)
Supplement: S1 Text — (DOCX) [file pone.0165508.s001.docx]

**S1. Summary of follow-up studies age 11-29**

Repeated tests of word decoding ability on the basis of 83 HR subjects (65 boys and 18 girls) and 79 LR subjects (62 boys and 17 girls) in 5th grade (age 11-12) and 9th grade (age 15-16) showed that the early decoding problems persisted over the years for the majority of HR subjects. By the end of the mandatory school period, in 9^th^ grade, 75% (71% of the boys and 89% of the girls) had not yet reached the average national standard of the 7^th^ grade (age 13-14) and only slightly more than half of the group, 58%, had attained the average standard of the 5^th^ grade (age 11-12). Thus, even though the subjects in the HR group managed to improve their word decoding skill, in some cases after having received increased hours of remedial instruction, most of them remained far behind their peers throughout the compulsory school years.

Towards the end of secondary school, interviews conducted with 44 HR and 36 LR subjects revealed significant differences with respect to their choice of study after having finished mandatory school. Only 10% of the students in the HR group opted for any of the broader theoretical study programs preparing for higher education, whereas more than half of the students in the LR group chose such a program. About 75% of the students in the HR group pursued vocational studies (83% had entered their first choice), as compared to 35% of the students in the LR group (91% had entered their first choice). A review of school grades by the end of secondary school also showed significant differences between the two groups in the core subjects Swedish, English, and mathematics. The percentage of students who failed to pass these subjects were significantly higher in the HR group, with the largest difference observed for English, where 38% failed compared to 9% in the LR group, and in mathematics, where 31% failed compared to 3% of the controls. None of the subjects in the control group failed in Swedish, whereas 14% of the HR subjects did.

At 29-30 years of age, 30 HR and 28 LR subjects were administered a battery of reading-related cognitive tests measuring phonological awareness, lexical accessibility, verbal memory, rapid automatized naming, word decoding, spelling, vocabulary, reading speed and reading comprehension. The results showed large differences between the groups, with the HR group performing substantially below the control group on all measures. The largest differences were found in tests of phonological awareness, spelling and word decoding, where the HR group performed on average 5 standard deviations below the mean of the control group. Overall, these results suggest that the difference between the two groups had not decreased in magnitude since the time of study entry, 20 years earlier. A questionnaire study prepared at the same age, which involved 60 HR and 73 LR subjects, further revealed that 17% of subjects in the HR group entered higher education after secondary school, compared to 50% in the LR group. It also showed that subjects in the HR group tended to settle down, marry and have children earlier in life than subjects in the control group. Subjects in the HR group also engaged in reading and writing activities less often, both at work and for leisure, and were more prone in general to downplay the importance of being able to read.
